# Automated and Highly Efficient Detection of Lignans in Plant-Derived Foods Based on Magnetic Solid-Phase Extraction Combined with UPLC

**DOI:** 10.3390/foods15203629

**Published:** 2026-10-12

**Authors:** Jiamei Zhu, Na Liang, Yang Wang, Shan Liang

**Affiliations:** Key Laboratory of Geriatric Nutrition and Health, School of Food and Health, Beijing Technology and Business University (BTBU), Beijing 100048, China; 18238370072@163.com (J.Z.); liangna2025@163.com (N.L.); wy777800@163.com (Y.W.)

**Keywords:** magnetic solid-phase extraction, metal–organic framework materials, lignans, UPLC, high-throughput detection

## Abstract

Lignans, a class of plant-derived polyphenolic compounds, exhibit significant bioactivities and serve as valuable functional food ingredients. However, conventional enrichment methods often suffer from inadequate selectivity and environmental incompatibility, limiting their practical application. In this study, the magnetic metal–organic framework Fe_3_O_4_@ZIF-67 was employed as an adsorbent for magnetic solid-phase extraction (MSPE). Coupled with ultra-performance liquid chromatography (UPLC), this method achieved rapid, selective, and high-efficiency enrichment of lignans—sesaminol, sesamin, and sesamolin. Method validation demonstrated excellent linearity (R^2^ ≥ 0.9999) over the concentration range of 0.5–100 μg/mL, acceptable recoveries (75.7–107.8%) with relative standard deviations (RSDs) ≤ 15%, and limits of detection (LODs) and limits of quantification (LOQs) in the ranges of 0.02–0.1 μg/mL and 0.09–0.25 μg/mL, respectively. Furthermore, an automated high-throughput detection system was developed through the integration of MSPE with UPLC, providing a simple and robust strategy for lignan quantification in functional food analysis.

## 1. Introduction

Growing consumer awareness of the nutritional composition of foods and their health implications has heightened interest in lignans—a class of beneficial polyphenolic nutrients widely distributed in the plant kingdom [1]. Structurally, lignans are diphenolic compounds characterized by oxidative coupling of two C6-C3 units at the β–β′ positions [2,3]. This structural class encompasses a diverse array of naturally occurring compounds, including sesamin, sesamolin, sesaminol, secoisolariciresinol, pinoresinol, and lariciresinol, along with their naturally occurring derivatives [4,5]. Major dietary sources of lignans include oilseeds (particularly flaxseed, sesame seed, and rapeseed), as well as legumes and select vegetables, notably members of the Brassicaceae family [6]. Functioning as phytoestrogens, lignans exhibit both weak estrogenic and anti-estrogenic activities, along with potent antioxidant properties. These characteristics enable their observed effects in cancer prevention, cardiovascular protection, and diabetes management [7,8,9]. Although lignans possess outstanding health-promoting effects, their trace content in plant tissues and complex matrix environments poses significant challenges for detection and analysis. These challenges hinder the development and application of lignans in the food industry, manifesting as several technical bottlenecks, including low enrichment efficiency, low detection accuracy, and a lack of unified detection standards [10,11]. Therefore, the development of robust, sensitive, and selective analytical methods for lignan detection and enrichment is critical to advancing their industrial applications.

Current separation and enrichment techniques for lignans in plant-derived foods predominantly rely on solvent extraction (SE) [12] and solid-phase extraction (SPE) [13], typically coupled with high-sensitivity analytical platforms (HPLC-MS, GC-MS) for accurate quantification of trace-level lignans in complex matrices. However, these methods suffer from high solvent consumption, insufficient selectivity, and operational complexity, motivating the development of more efficient and environmentally sustainable alternatives [14,15]. In this context, magnetic solid-phase extraction (MSPE) has emerged as a promising alternative [16]. Relative to conventional techniques, MSPE offers lower operational costs, improved reusability, enhanced environmental sustainability, and compatibility with automated analytical systems [17]. The utility of MSPE has been demonstrated in diverse plant-derived food and natural product analysis scenarios, including the enrichment of multiple flavonoids from herbal plant matrices using Fe_3_O_4_@ZIF-67 composite adsorbent [18] and the simultaneous quantification of phenolic bioactive components in oilseed matrices with Fe_3_O_4_-r-GO magnetic adsorbent [19]. In addition, Fe_3_O_4_@DUT-5-DABA composite adsorbents have been employed to extract lignans from Schisandra chinensis [20]. Although these studies demonstrated the feasibility of magnetic MOF-based adsorbents for lignan extraction, both focused solely on lignans in a single matrix [20,21]. However, the application of Fe_3_O_4_@ZIF-67 to the simultaneous enrichment and determination of sesaminol, sesamin, and sesamolin in diverse plant-derived food matrices remains insufficiently investigated.

The key to enriching lignans using MSPE technology lies in exploring highly efficient magnetic adsorbents; however, conventional adsorbent materials commonly display suboptimal adsorption kinetics, chiefly resulting from their low specific surface area and inadequate availability of active adsorption sites. To address these limitations, nanoscale adsorbents have been developed, which exhibit exceptionally high specific surface areas and enhanced selectivity toward small organic molecules [22]. Among these, metal–organic frameworks (MOFs) have emerged as a particularly promising class. MOFs are crystalline, porous materials constructed via the coordination of metal ions or clusters with multitopic organic linkers [23], which endow them with ultrahigh specific surface areas, precisely tunable pore structures, and diverse, chemically modifiable functionalities [24,25]. Zeolitic imidazolate frameworks (ZIFs) represent a structurally distinctive and widely studied subclass of MOFs [26,27]. ZIF-67, a prototypical member of this family, is synthesized via the coordination of cobalt (II) ions with 2-methylimidazolate linkers in methanol. Benefiting from the above structural features, ZIF-67 has been extensively adopted as an adsorbent for diverse analytical scenarios, including the adsorption of polyphenols in various aqueous matrices [28] and the enrichment of antibiotics in milk [29]. This material exhibits several advantageous attributes: (1) well-defined pore structures with suitable pore and window dimensions; (2) robust chemical and thermal stability; (3) coordinatively unsaturated cobalt (II) sites that remain accessible within the framework, enabling direct coordination with adsorbate molecules; and (4) negligible solubility in water and common organic solvents [30,31]. Collectively, these attributes render ZIF-67 a highly promising adsorbent for the selective capture of organic molecules. Given that lignans are typically present at trace levels in complex plant matrices and possess multiple functional groups amenable to coordination interactions, ZIF-67 holds significant potential for the selective and efficient extraction of this target class.

**Scheme 1:**
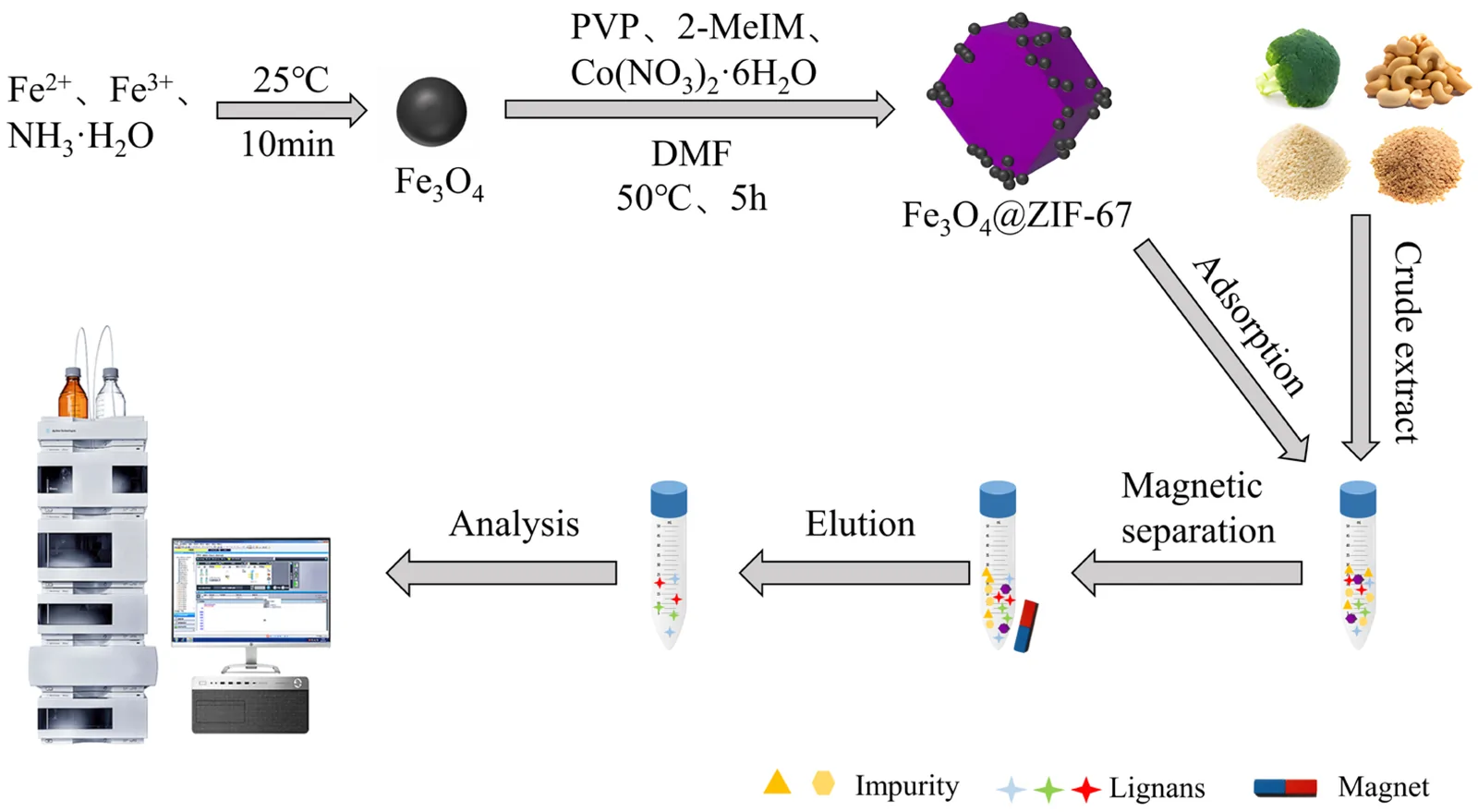
Preparation process of Fe_3_O_4_@ZIF-67 composite material and the MSPE procedure for the determination of lignans.

In this study, a magnetic metal–organic framework Fe_3_O_4_@ZIF-67 was prepared by the solvothermal method and was used as an adsorbent for the enrichment of lignans. The magnetic solid-phase extraction (MSPE) process based on Fe_3_O_4_@ZIF-67 was integrated into an automated magnetic extraction platform, and combined with ultra-high-performance liquid chromatography (UPLC), a method for simultaneously quantifying sesamin, sesaminol, and sesamolin in plant-derived food matrices was developed (Scheme 1). Under optimized conditions, this method demonstrated excellent analytical performance in terms of extraction recovery rate, sensitivity, and repeatability and could perform high-throughput automatic extraction. These results confirmed that the proposed method is applicable for the determination of lignans in complex food matrices.

## 2. Materials and Methods

### 2.1. Materials and Chemicals

Ferric (III) chloride hexahydrate (FeCl_3_·6H_2_O) and ferrous (II) chloride tetrahydrate (FeCl_2_·4H_2_O) were purchased from Sinopharm Chemical Reagent Co., Ltd. (Shanghai, China). Ammonia solution (NH_3_·H_2_O), trisodium citrate dihydrate, cobalt nitrate hexahydrate (Co(NO_3_)_2_·6H_2_O), and 2-methylimidazole were all analytical reagent grade and purchased from Shanghai Macklin Biochemical Co., Ltd. (Shanghai, China). Polyvinylpyrrolidone (PVP) was purchased from Beijing Solarbio Science and Technology Co., Ltd. (Beijing, China). Anhydrous ethanol and N,N-dimethylformamide (DMF, ≥99.5%) were purchased from Tianjin Fuchen Chemical Co., Ltd. (Tianjin, China). Methanol and acetonitrile were supplied by Beijing Mreda Technology Co., Ltd. (Beijing, China).

Reference standards of sesaminol, sesamin, and sesamolin (purity ≥ 98%) were purchased from Shanghai Yuanye Biotechnology Co., Ltd. (Shanghai, China). Structural formulae of the three lignans are presented in Figure S1. Individual stock standard solutions (1 g/L) were prepared by dissolving each pure compound in methanol, and the resulting solutions were stored at −20 °C in the dark to minimize photodegradation. Working standard solutions were then prepared by serial dilution of each stock solution with methanol and were freshly prepared before analysis.

### 2.2. Instrumentation and UPLC Conditions

Transmission electron microscopy (TEM) images were obtained with a JEM-F200 microscope (JEOL Ltd., Tokyo, Japan). Energy dispersive X-ray spectroscopy (EDX) spectra were recorded on a JED-2300T spectrometer (JEOL Ltd., Tokyo, Japan). Fourier-transform infrared (FT-IR) spectra were recorded with a Nicolet iS50 spectrometer (Thermo Fisher Scientific, Waltham, MA, USA). X-ray diffraction (XRD) patterns were obtained on a Shimadzu XRD-6100 diffractometer (Shimadzu, Kyoto, Japan). Magnetic properties were measured on a Lake Shore 8604 vibrating sample magnetometer (VSM) (Lake Shore Cryotronics, Westerville, OH, USA). Thermogravimetric analysis (TGA) was performed with a NETZSCH TG 209F3 thermogravimetric analyzer (NETZSCH-Gerätebau GmbH, Selb, Germany). The Brunauer–Emmett–Teller (BET) specific surface area and pore parameters were calculated from nitrogen adsorption–desorption isotherms measured by a Quantachrome Autosorb-iQ analyzer (Quantachrome Instruments, Boynton Beach, FL, USA). High-throughput magnetic solid-phase extraction of target lignans was implemented with the Purifier HT nucleic acid extraction and purification system (Genfine Biotech, Changzhou, China) (Figure S2).

Lignan analysis was performed using an Agilent 1290 Infinity II ultra-performance liquid chromatography system equipped with a diode-array detector (DAD) (Agilent Technologies Co., Ltd., Beijing, China). Chromatographic separation was carried out on an Agilent InfinityLab Poroshell 120 SB-C18 column (3.0 mm × 100 mm, 2.7 μm) kept at 35 °C. Methanol was designated as mobile phase A and ultrapure water as mobile phase B. The gradient elution program was employed as follows: 0 min, 30% A; 3 min, 60% A; 6.5 min, 80% A; 8–10 min, 100% A. The flow rate was fixed at 0.4 mL/min, and the injection volume was 5 μL. Detection was conducted at 285 nm with a bandwidth of 4 nm. Sesaminol, sesamin, and sesamolin were assigned by comparing their retention times with those of authentic standards analyzed under identical chromatographic conditions. A representative chromatogram of the mixed standards is provided in Figure S3.

### 2.3. Preparation of Fe_3_O_4_ Nanoparticles

Fe_3_O_4_ nanoparticles were synthesized via a modified co-precipitation method based on a previously reported procedure [32]. Briefly, 1.329 g of FeCl_3_·6H_2_O and 0.489 g of FeCl_2_·4H_2_O were accurately weighed and dissolved in a three-necked round-bottom flask with 20 mL of ultrapure water and 30 mL of anhydrous ethanol under continuous nitrogen protection to avoid oxidation of ferrous ions. The reaction system was purged with nitrogen for 15 min to remove dissolved oxygen fully, after which 3 mL of aqueous ammonia solution (25–28%) was quickly injected into the mixed solution, and the reaction system was mechanically stirred at room temperature for 10 min. The resulting Fe_3_O_4_ nanoparticles were collected by magnetic separation with an external magnet. The nanoparticles were first washed once with 1% aqueous sodium citrate solution and then rinsed three times with ultrapure water. Finally, the purified nanoparticles were redispersed in ultrapure water for subsequent use.

### 2.4. Preparation of Fe_3_O_4_@ZIF-67

Add PVP at a mass ratio of 5:1 to the above Fe_3_O_4_ nanoparticle dispersion solution. Then, shake it at 25 ± 2 °C for 20 h to achieve the coating of PVP. The obtained Fe_3_O_4_@PVP nanoparticles are collected using an external magnet, washed twice with ultrapure water, and once with DMF to remove the unbound PVP. They are then redispersed in DMF. Next, uniformly disperse 0.30 g of Fe_3_O_4_@PVP nanoparticles into 80 mL of methanol and perform 10 minutes of ultrasonic treatment. Then, 4.6536 g of Co(NO_3_)_2_·6H_2_O dissolved in 80 mL of methanol was introduced into the dispersion, and the mixed solution was ultrasonicated for another 15 min. After ultrasonication, the suspension was transferred to a water bath at 50 °C and stirred at 800 rpm for 30 min. Separately, 2-methylimidazole (5.1266 g) was dissolved in 160 mL of methanol. The resulting solution was then rapidly added to the heated mixture under continuous stirring, and the reaction was maintained for 5 h. Following the reaction, the obtained composites were magnetically separated using an external magnet, alternately washed with absolute ethanol and ultrapure water three times, and subsequently dried at 60 °C for 24 h [33].

### 2.5. Sample Preparation

Broccoli, cashew nuts, sesame seeds, and flaxseeds were purchased from a local supermarket (Beijing, China). The plant samples were thoroughly washed with ultrapure water to remove surface contaminants, ground, sieved through a 60-mesh sieve, and stored at 4 °C before use. For sample extraction, 2.0 g of the sieved sample was transferred to a centrifuge tube, mixed with 10 mL of methanol, and vortexed for 2 min. After centrifugation at 5000 rpm for 5 min, the supernatant was collected and filtered through a 0.22 μm nylon membrane syringe filter for the following MSPE procedure.

### 2.6. MSPE Procedure

Scheme 1 describes the MSPE procedure. In detail, 300 mg of Fe_3_O_4_@ZIF-67 was added to a 10 mL centrifuge tube containing 2 mL of sample solution. The mixture was shaken for 10 min to complete the adsorption of target lignans. The analyte-loaded magnetic composites were separated from the supernatant using an external magnet. Afterwards, 8 mL of DMF was added to the harvested adsorbents, and vortex mixing was performed for 6 min to elute the target analytes. Following magnetic separation, the DMF eluate was collected and blown down to near dryness under a mild nitrogen flow. Finally, it was dissolved in 1 mL of methanol solution, filtered through a 0.22 μm nylon membrane syringe filter, and analyzed using UPLC.

### 2.7. Adsorption Performance Evaluation of Fe_3_O_4_@ZIF-67

To evaluate the adsorption performance of the as-prepared magnetic composites toward lignans, adsorption kinetic experiments were conducted. Specifically, 300 mg of Fe_3_O_4_@ZIF-67 was dispersed in 2 mL of a mixed standard solution containing sesaminol, sesamin, and sesamolin (each at 2 mg/L), and the suspension was vortexed at room temperature for varying time intervals (1, 2, 4, 6, and 10 min). After magnetic separation with an external magnet, the supernatant was collected and analyzed by UPLC. The adsorption capacity was calculated according to Equation (1) [34]:

q = (C_0_ − C_e_)V/m (1)

where q (mg/g) is the adsorption capacity of the adsorbent, C_0_ (mg/L) is the initial concentration of lignans, C_e_ (mg/L) is the equilibrium concentration of lignans in solution, V (mL) is the adsorption volume of lignans, and m (g) is the mass of the adsorbent used.

The adsorption kinetic data were fitted to the pseudo-first-order [35] and pseudo-second-order kinetic models [36] to characterize the adsorption kinetics of the three lignans on Fe_3_O_4_@ZIF-67. These models are expressed by Equations (2) and (3), respectively:

log(q_e_ − q_t_) = logq_e_ − (k_1_/2.303)t (2)

(3) $$t/q_{t}=1/k_{2}q_{e}^{2}+(1/q_{e})t$$

where q_e_ (mg/g) denotes the equilibrium adsorption capacity of lignans, q_t_ (mg/g) is the adsorption capacity at time t (min), and k_1_ (min^−1^) and k_2_ (g/(mg·min)) represent the rate constants of the pseudo-first-order and pseudo-second-order kinetic models, respectively.

To further clarify the intraparticle diffusion pathway of lignans onto Fe_3_O_4_@ZIF-67, the Weber-Morris intraparticle diffusion model was applied [37]. This diffusion process consists of three sequential stages: first, lignan molecules migrate from the bulk solution to the adsorbent outer surface; then, the target compounds enter the pore channels and undergo intraparticle diffusion inside the material; and finally attach to the internal active sites until equilibrium. The model calculation is based on Equation (4):

q_t_ = k_3_t^1/2^ + I (4)

k_3_(mg/(g·min^1/2^)) denotes the intraparticle diffusion rate constant, and I reflects the boundary layer resistance.

### 2.8. Method Validation

To validate the applicability of the established MSPE-UPLC method for lignan analysis, the method was systematically validated in terms of linearity, limit of detection (LOD), limit of quantification (LOQ), system suitability, accuracy, and precision.

Under the optimized experimental conditions, six calibration standards of mixed lignans (0.5, 8, 20, 40, 80, and 100 μg/mL) were prepared in methanol, and linearity was evaluated by plotting peak area versus corresponding concentration to calculate the coefficient of determination (R^2^). The LOD and LOQ were determined based on signal-to-noise (S/N) ratios of 3 and 10, respectively. An 8 μg/mL mixed lignan reference solution was utilized to verify the system suitability of the established UPLC method. The evaluation indices consisted of the relative standard deviations (RSD) of retention time and peak area after six consecutive injections, together with the symmetry factor of each chromatographic peak. Accuracy was evaluated by recovery experiments: different blank samples were spiked with mixed lignan standards at three concentration levels (low, medium, and high), and the recoveries were used to assess method accuracy. The recovery of each lignan was calculated following Equation (5):

(5) $$\mathrm{Recovery}(\%)=\frac{C(\mathrm{spiked})-C(\mathrm{matrix})}{C(\mathrm{standard})}\times 100\%$$

where C(spiked), C(matrix), and C(standard) represent the concentrations of spiked samples, unspiked samples, and standard solution, respectively. The precisions were expressed as intra- and inter-day relative standard deviation (RSD), which were obtained via testing the same samples six times in one day and analyzing the same samples for three contiguous days, respectively.

### 2.9. High-Throughput Magnetic Extraction Procedure

The operating program of the fully automated nucleic acid extractor was reprogrammed to satisfy the unique enrichment demands for lignans, enabling its use as a dedicated pretreatment platform. The modified system can process up to 96 samples in parallel per batch. Under the optimized MSPE conditions, the system automated the principal extraction steps, including magnetic adsorbent collection, analyte adsorption, and elution, for lignans in plant-derived food extracts. The extraction process consisted of four sequential steps: magnetic adsorbent collection, analyte binding, solvent elution, and waste disposal, with operational parameters detailed in Table S1. First, the magnetic bar parameters were programmed to collect 300 mg of magnetic adsorbent. Subsequently, a 10 min shaking period was applied to promote sufficient binding between lignans and the adsorbent. Following this, 8 mL DMF was added for vortex elution lasting 6 min to desorb the collected lignans. Finally, the waste solution was discharged after elution. The resulting eluate was collected and blown down to near dryness under a mild nitrogen flow. Finally, it was dissolved in 1 mL of methanol solution, filtered through a 0.22 μm nylon membrane syringe filter, and analyzed using UPLC.

### 2.10. Statistical Analysis

All data were expressed as the mean ± standard deviation from three independent experiments. One-way analysis of variance (ANOVA) was performed with IBM SPSS Statistics 20.0 (IBM Corp., Chicago, IL, USA) to assess inter-group differences. Differences with *p* < 0.05 were considered statistically significant.

## 3. Results and Discussion

### 3.1. Preparation and Characterization of Fe_3_O_4_@ZIF-67

Scheme 1 illustrates the synthesis of Fe_3_O_4_@ZIF-67 and its application in the MSPE of lignans. To confirm successful adsorbent synthesis and evaluate its structural and functional properties, Fe_3_O_4_@ZIF-67 was systematically characterized by transmission electron microscopy (TEM), energy-dispersive X-ray spectroscopy (EDX), Fourier transform infrared (FT-IR) spectroscopy, X-ray diffraction (XRD), vibrating sample magnetometer (VSM), thermogravimetric analysis (TGA), and N_2_ adsorption–desorption experiments.

**Figure 1:**
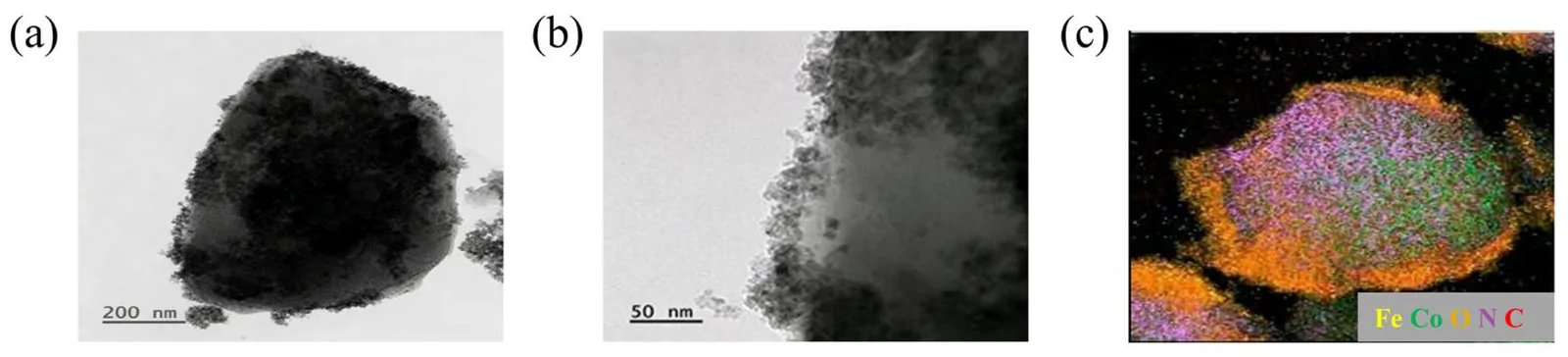
(**a**,**b**) TEM image of Fe_3_O_4_@ZIF-67; (**c**) EDX image of Fe_3_O_4_@ZIF-67.

TEM was used to characterize the morphology and particle size distribution of the synthesized Fe_3_O_4_@ZIF-67 composite. As shown in Figure 1a,b, the material consisted predominantly of cubic nanoparticles with diameters ranging from 500 to 600 nm. High-resolution TEM (HR-TEM) images further revealed that Fe_3_O_4_ nanoparticles were homogeneously dispersed on the ZIF-67 surface, providing preliminary evidence for the successful fabrication of the Fe_3_O_4_@ZIF-67 composite. Furthermore, EDX elemental mapping was performed to analyze the elemental composition and spatial distribution of the composite (Figure 1c). The mapping results identified the presence of C, N, O, Co, and Fe elements. C, N, and Co exhibited uniform distribution across the entire ZIF-67 particle framework, whereas Fe and O signals were predominantly enriched at the periphery of nanoparticles [38]. This unique elemental distribution matched well with the HR-TEM observation that Fe_3_O_4_ nanoparticles were immobilized on the outer surface of ZIF-67, which further validated the successful preparation of the Fe_3_O_4_@ZIF-67 composite.

**Figure 2:**
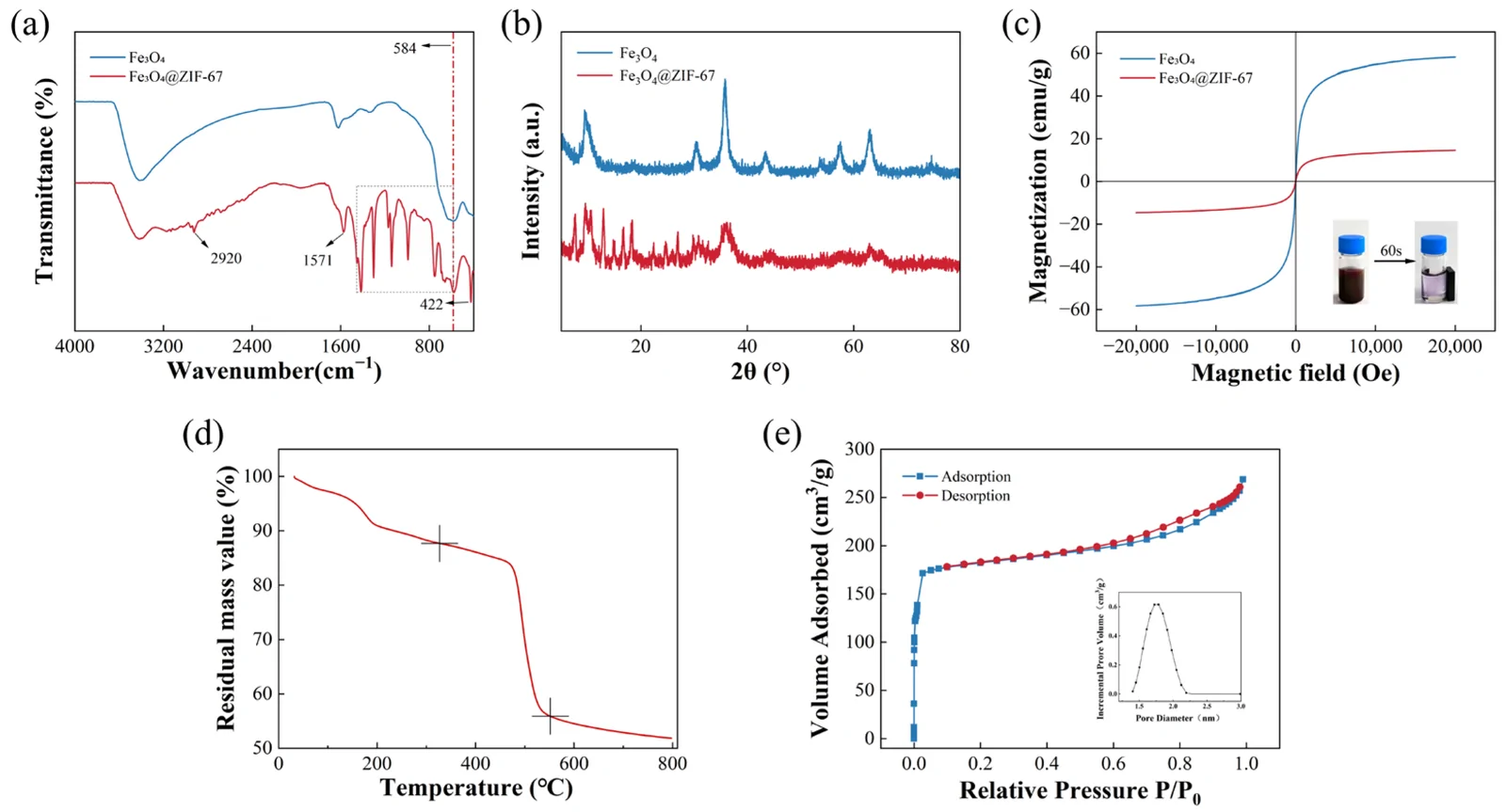
(**a**) FT-IR spectra of Fe_3_O_4_ and Fe_3_O_4_@ZIF-67; (**b**) XRD spectra of Fe_3_O_4_ and Fe_3_O_4_@ZIF-67; (**c**) VSM curves of Fe_3_O_4_ and Fe_3_O_4_@ZIF-67; (**d**) TGA curves of Fe_3_O_4_ and Fe_3_O_4_@ZIF-67; (**e**) N_2_ adsorption–desorption isotherms of Fe_3_O_4_@ZIF-67 with an inset of pore size distribution.

The functional groups and chemical bonding environment of the magnetic nanocomposite were characterized by FT-IR spectroscopy (Figure 2a). A characteristic absorption band at 422 cm^−1^ was assigned to Co-N stretching vibrations within the ZIF-67 framework [39], and the band at 584 cm^−1^ was attributed to the Fe-O stretching vibration from the Fe_3_O_4_ component [40]. Within the wavenumber range of 600 cm^−1^ to 1500 cm^−1^, distinct absorption bands corresponding to characteristic vibrational modes of the imidazole ring were observed, confirming the incorporation of the imidazole moiety in the ZIF-67 framework. Additional absorption bands at 1571 cm^−1^ and 2920 cm^−1^ were assigned to the C=N stretching vibration and aromatic C-H stretching vibration of 2-methylimidazole, respectively [41,42]. Notably, the intensity of the Fe-O vibrational band diminished following ZIF-67 modification relative to that of bare Fe_3_O_4_ nanoparticles, which was reasonably attributed to the strong interfacial electronic interactions between Fe_3_O_4_ and the ZIF-67 framework. To further elucidate structural characteristics and assess phase purity, Fe_3_O_4_ and Fe_3_O_4_@ZIF-67 were subjected to XRD analysis. As shown in Figure 2b, Fe_3_O_4_ displayed characteristic diffraction peaks at 2θ = 30.0°, 35.4°, 43.0°, 53.4°, 57.0°, and 62.5°, which were indexed to the (220), (311), (400), (422), (511), and (440) crystallographic planes, respectively [43]. For Fe_3_O_4_@ZIF-67, multiple sharp new peaks appeared at low angles of 2θ = 12.78°, 14.84°, 16.59°, and 18.23°, which match the typical crystalline diffraction signals of ZIF-67 [44]. Meanwhile, the characteristic peaks of Fe_3_O_4_ were still observable in the composite with obviously reduced intensity, with no extraneous phases detected. Collectively, the combination of FT-IR and XRD results confirmed that the crystalline structure of Fe_3_O_4_ remained intact during composite fabrication and that a phase-pure Fe_3_O_4_@ZIF-67 composite was successfully synthesized.

The magnetic properties of Fe_3_O_4_ and Fe_3_O_4_@ZIF-67 were evaluated by VSM. As shown in Figure 2c, the saturation magnetization values were determined to be 58.23 emu·g^−1^ for Fe_3_O_4_ and 14.59 emu·g^−1^ for Fe_3_O_4_@ZIF-67. The magnetization curves of both materials exhibited negligible coercivity and remanence, confirming their excellent superparamagnetic behavior. Although ZIF-67 incorporation reduced the saturation magnetization of the composite relative to pristine Fe_3_O_4_, the retained magnetization remained sufficient to enable efficient magnetic separation. Experimental results demonstrated that the Fe_3_O_4_@ZIF-67 nanocomposite could be completely separated from aqueous suspension within 60 s upon application of an external magnetic field, confirming suitability for practical magnetic separation applications.

TGA was performed to monitor the mass change in the sample against temperature and characterize its thermal stability. As shown in Figure 2d, the first mass-loss step (30–300 °C) was accompanied by a weight loss of 12.03%, which was attributed primarily to the physical desorption of adsorbed water and residual solvent molecules [45]. The second mass-loss step (300–550 °C) brought about a significant mass loss of 34.34%, arising from the thermal decomposition of the ZIF-67 framework and the concomitant collapse of its porous structure [46]. At 800 °C, 53.63% of the initial mass was retained, which might be attributed to the inorganic residues produced during the thermal decomposition of Fe_3_O_4_ and ZIF-67. Collectively, these data indicated that the Fe_3_O_4_@ZIF-67 composite remained structurally intact up to approximately 300 °C, demonstrating that the material possesses adequate thermal stability for applications at operating temperatures below 300 °C.

To further characterize the specific surface area and pore structural parameters of the magnetic composite, N_2_ adsorption–desorption isotherms were measured. As shown in Figure 2e, according to IUPAC classification, the adsorption isotherm of Fe_3_O_4_@ZIF-67 corresponded to a Type IV curve [47], which indicated that the material contained abundant micropores accompanied by a small number of interparticle mesopores. BET calculations showed that the Fe_3_O_4_@ZIF-67 composite possessed a high specific surface area of 759.80 m^2^/g and a total pore volume of 0.28 cm^3^/g. Density functional theory (DFT) modeling of the N_2_ adsorption isotherm determined an average pore diameter of 1.79 nm. The large specific surface area and well-developed porous structure provided numerous adsorption-active sites for lignan molecules. Meanwhile, the micropore-dominated pore structure endowed the material with a favorable size-selective adsorption ability toward lignan molecules.

### 3.2. MSPE Parameter Optimization

To determine the optimal extraction conditions, the effects of key experimental parameters—namely, adsorbent dosage, extraction time, and desorption solvent—on the extraction efficiency of the three target lignans (sesaminol, sesamin, and sesamolin) were systematically investigated and optimized via a one-variable-at-a-time (OVAT) strategy.

#### 3.2.1. Effect of Adsorbent Dosage

**Figure 3:**
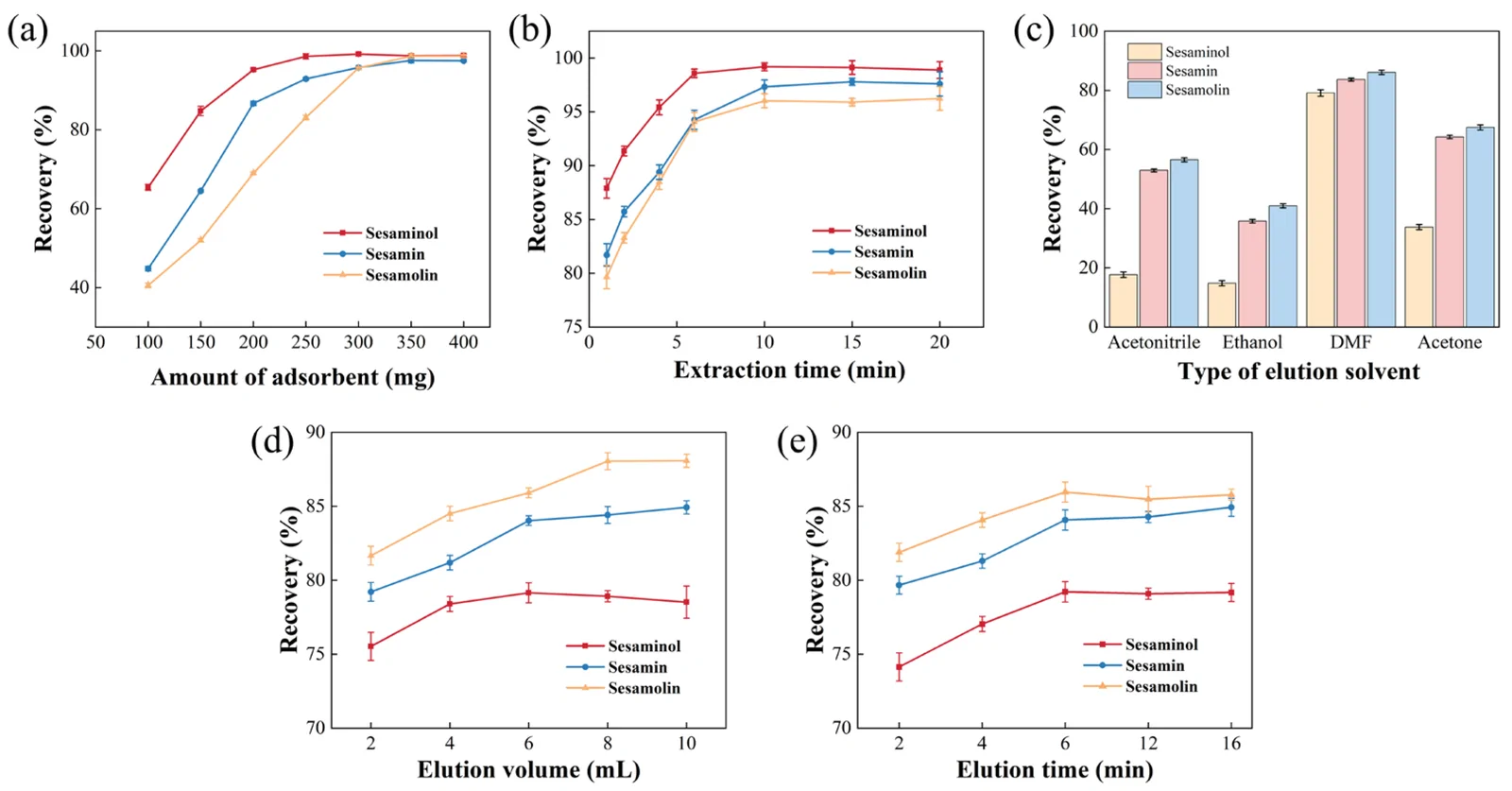
Effect of (**a**) the amount of adsorbent; (**b**) extraction time; (**c**) elution solvent type; (**d**) elution volume; (**e**) elution time on extraction efficiency with Fe_3_O_4_@ZIF-67.

Adsorbent dosage directly affected the adsorption capacity for target analytes. In this study, the influence of adsorbent dosage on the adsorption efficiency of the three lignans was systematically evaluated. As indicated in Figure 3a, the adsorption of lignans by Fe_3_O_4_@ZIF-67 increased significantly as the adsorbent dosage increased and reached a plateau at 300 mg. No further significant enhancement in adsorption uptake was detected when the dosage was further increased. Therefore, 300 mg was adopted as the optimal adsorbent dosage for subsequent experiments.

#### 3.2.2. Effect of Extraction Time

Extraction time between the adsorbent and target analytes was a critical parameter governing adsorption performance. The effect of extraction time on the adsorption efficiency of Fe_3_O_4_@ZIF-67 toward the three lignans was systematically assessed. As shown in Figure 3b, the adsorption efficiency increased markedly within the first 10 min, after which the adsorption rate decreased significantly. This phenomenon was attributed to the gradual occupation of active sites on the adsorbent surface by lignan molecules until adsorption equilibrium was achieved. Consequently, 10 min was adopted as the optimal extraction time for lignan adsorption.

#### 3.2.3. Effect of Elution Solvent Type

The polarity of organic solvents was considered a critical determinant of their elution efficacy for target analytes. To identify the optimal eluent, four representative polar solvents (acetonitrile, ethanol, DMF, and acetone) were comparatively evaluated for their elution efficiency toward the three lignans. As shown in Figure 3c, DMF achieved the highest recoveries for all detected compounds and exhibited better elution ability than the other solvents. This superior performance was attributed to the strong dipole–dipole and hydrogen-bonding interactions between the highly polar DMF molecules and the polar functional groups (e.g., hydroxyl groups in sesaminol) of the lignans. This significantly enhanced their elution from the adsorbent. Accordingly, DMF was selected as the optimal elution solvent for subsequent experiments.

#### 3.2.4. Effect of Elution Volume

The volume of elution solvent critically influenced the elution efficiency of target analytes from the adsorbent. Insufficient volume compromised quantitative recovery, whereas excessive volume brought extra environmental risks and increased subsequent treatment workload. In this study, the elution efficiency of the three lignans pre-adsorbed onto Fe_3_O_4_@ZIF-67 was evaluated using incremental volumes of DMF: 2, 4, 6, 8, and 10 mL. As shown in Figure 3d, no obvious improvement in elution efficiency was obtained when the DMF dosage exceeded 8 mL. Accordingly, 8 mL of DMF was determined as the optimal elution volume, which achieved a good balance among satisfactory recovery, solvent saving, and experimental operability.

#### 3.2.5. Effect of Elution Time

The influence of elution time on analyte recovery was systematically investigated across a series of time intervals: 2, 4, 6, 12, and 16 min. As shown in Figure 3e, under optimal eluent conditions, a recovery efficiency exceeding 70% was achieved within 2 min. Elution equilibrium was reached at 6 min, beyond which no significant increase in recovery was observed. Accordingly, 6 min was confirmed as the optimal elution time to guarantee high analysis efficiency and stable detection performance.

### 3.3. Adsorption Performance of Fe_3_O_4_@ZIF-67

Under the optimized MSPE conditions, Fe_3_O_4_@ZIF-67 exhibited favorable enrichment performance toward the three lignans (Figure S4). π–π interactions between the imidazolate linkers of ZIF-67 and the aromatic moieties of the target lignans may contribute to this adsorption behavior [48]. As a representative cobalt-based MOF, ZIF-67 possesses a highly porous, crystalline three-dimensional framework constructed by coordination bonds between Co^2+^ centers and 2-methylimidazole ligands. The hierarchically porous structure of Fe_3_O_4_@ZIF-67 (dominated by micropores with a tiny amount of interparticle mesopores) facilitates the adsorption of small-molecule lignans. Specifically, the polar hydroxyl group of sesaminol formed favorable interactions with surface polar sites primarily through orientation and induction forces, thereby enhancing binding affinity [21]. Considering the predominantly nonpolar structures of sesamin and sesamolin, their adsorption may be associated with dispersion interactions with the sorbent surface. In addition, the surface characteristics and hierarchical porosity of Fe_3_O_4_@ZIF-67 may provide accessible adsorption sites for these lignans.

Adsorption kinetic models were applied to describe the adsorption behavior of the three lignans on Fe_3_O_4_@ZIF-67. The pseudo-second-order kinetic model (Figure S5b) produced a higher coefficient of determination (R^2^ ≥ 0.9994) compared with the pseudo-first-order model (Figure S5a) (R^2^ ≥ 0.9741). Furthermore, the calculated equilibrium adsorption capacity (q_e_, cal) derived from the pseudo-second-order model exhibited closer agreement with the experimentally determined value (q_e_, exp) (Table S2). Accordingly, the pseudo-second-order model provided a better fit to the adsorption kinetic data of the three lignans.

The intraparticle diffusion model was applied to interpret the kinetic data, and the corresponding fitted plots are presented in Figure S5c, with model parameters summarized in Table S3. The fitted straight line from intraparticle diffusion regression displayed a low coefficient of determination and did not cross the origin of coordinates. This result revealed obvious boundary layer resistance, confirming that pore diffusion alone could not dominate the whole adsorption rate. Accordingly, the overall adsorption kinetics of the three lignans onto Fe_3_O_4_@ZIF-67 were governed by both intraparticle diffusion and liquid-film diffusion.

### 3.4. Reproducibility and Stability of Fe_3_O_4_@ZIF-67

**Figure 4:**
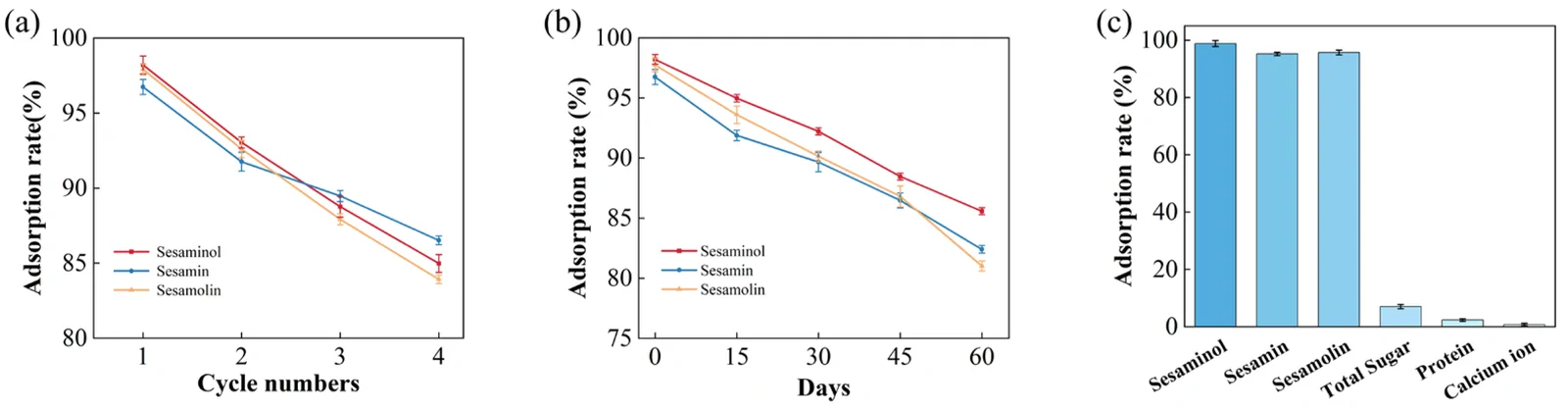
Effect of (**a**) cycle number; (**b**) storage day; (**c**) interferents on sesaminol, sesamin, and sesamolin adsorption from Fe_3_O_4_@ZIF-67.

Reusability and structural stability are vital indicators to evaluate adsorbents for lignan enrichment in practical sample pretreatment. After four consecutive adsorption–desorption cycles using the same batch of Fe_3_O_4_@ZIF-67, the material retained more than 80% of its initial adsorption capacity for all three target lignans, and only a slight decline in extraction efficiency was observed (Figure 4a). Before each cycle, the Fe_3_O_4_@ZIF-67 adsorbent was fully regenerated by successive rinsing with anhydrous ethanol and ultrapure water to guarantee stable performance in repeated recycling. Notably, the adsorbent still maintained over 80% of the initial adsorption capacity for the three lignans after 60 days of storage at room temperature, which proved its outstanding long-term structural and functional stability (Figure 4b). Finally, the material’s anti-interference performance was tested by adding typical interfering substances (glucose, proteins, metal ions) into simulated food matrices to simulate actual sample environments. Fe_3_O_4_@ZIF-67 exhibited high adsorption rates for sesaminol, sesamin, and sesamolin, whereas the adsorption rates of the tested interferents were considerably lower (Figure 4c). The interference experiment showed substantially higher adsorption of the three lignans than of the tested total sugar, protein, and calcium ions, indicating preferential adsorption of the target analytes under the tested conditions. Collectively, these findings substantiated the material’s strong reusability, favorable reproducibility, and excellent matrix anti-interference ability.

### 3.5. Method Validation

Method validation conducted under the optimized MSPE-UPLC conditions demonstrated excellent linearity, sensitivity, accuracy, and precision. As summarized in Table 1, the three lignans exhibited good linear responses over the concentration range of 0.5–100 μg/mL, with the correlation coefficients R^2^ > 0.9999. The LOD and LOQ were determined at signal-to-noise ratios of 3 and 10, in the ranges of 0.02–0.10 and 0.09–0.25 μg/mL, respectively, which confirmed the sensitivity of the method. Instrument stability and analytical reproducibility were evaluated through system suitability testing. Six consecutive replicate injections of mixed standard solutions produced RSDs not exceeding 1% for retention time and 5% for peak area (Table S4). As shown in Table 2 for broccoli, cashew nut, and flaxseed and in Table S5 for sesame, spiked recovery assays at three concentration levels (low, medium, and high) afforded average recoveries from 75.7% to 107.8%, with intra-day and inter-day RSD below 15%. These results confirmed the favorable accuracy and precision of the established analytical method.

### 3.6. Comparison of Fe_3_O_4_@ZIF-67 with Existing Extraction Methods

The analytical performance of the developed MSPE-UPLC method was systematically validated by comparison with previously reported lignan determination approaches. As presented in Table 3, the method delivered high recoveries of 79.16–83.12% for all three target lignans, which verified its excellent extraction efficiency and robust quantitative performance. A prominent advantage originated from the use of the magnetic Fe_3_O_4_@ZIF-67 adsorbent. Its intrinsic superparamagnetic property enables rapid magnetic separation, eliminating the need for centrifugation and filtration, simplifying sample pretreatment procedures, and rendering the method readily compatible with high-throughput automated sample pretreatment systems.

### 3.7. High-Throughput Automated Experimental Validation

A high-throughput extraction method for lignans was developed through systematic optimization of key operational parameters. Comparative evaluation of three lignan reference standards showed no obvious discrepancy in extraction efficiency between the automated workflow and the traditional manual operation (Figure S6). Moreover, the automated system yielded RSDs below 3% across all recovery experiments, demonstrating that its repeatability and stability were equivalent to those of manual pretreatment. This finding further verified that the proposed method applies to large-scale and routine laboratory analysis.

**Figure 5:**
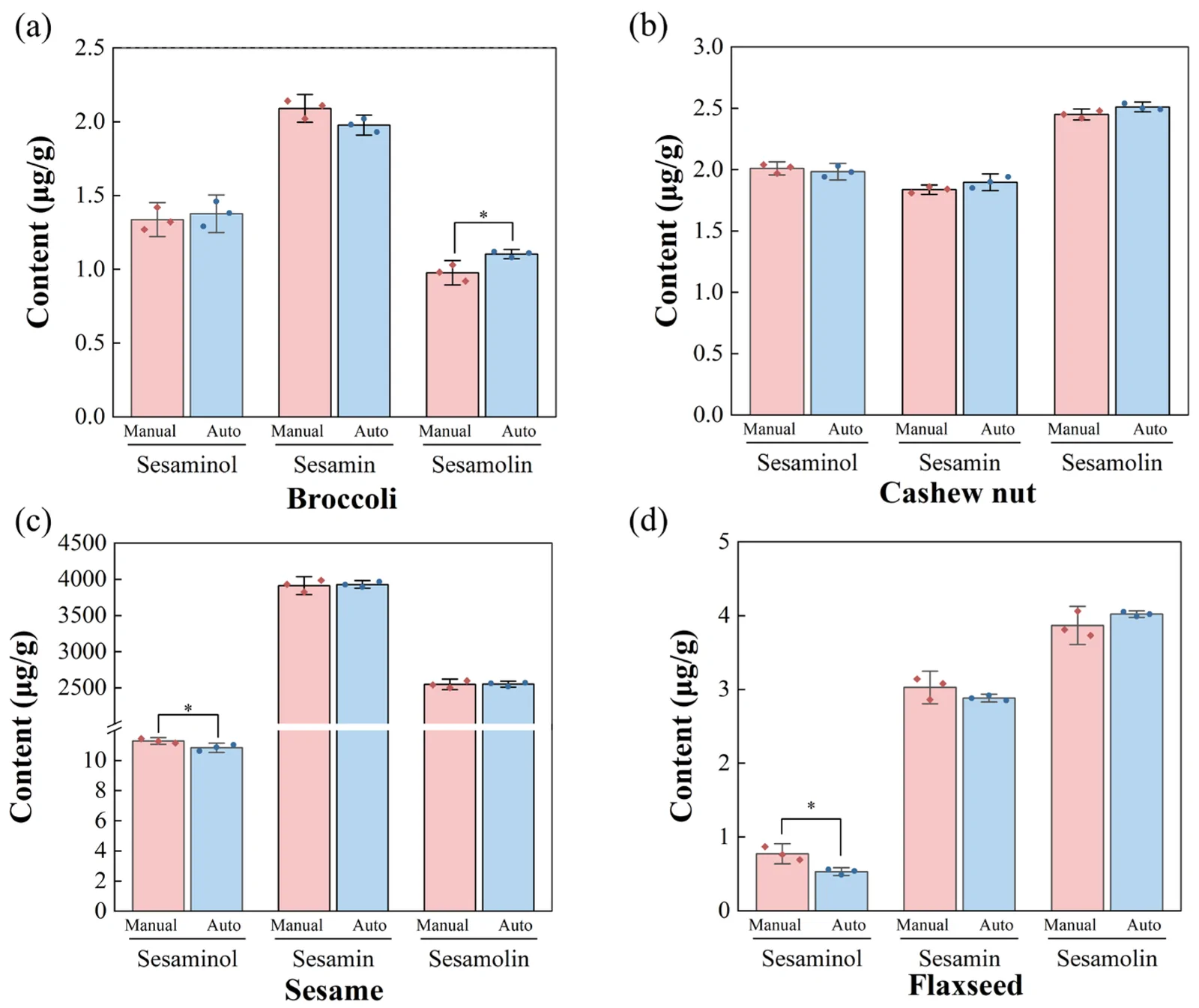
Comparison of the contents of three lignans in (**a**) broccoli, (**b**) cashew nuts, (**c**) sesame, and (**d**) flaxseed between automatic extraction and manual operation. The results are expressed as the mean ± standard deviation (*n* = 3). “*“ represents a significant difference at *p* < 0.05 between automatic extraction and manual operation.

Following method validation under automated conditions, the developed method was applied to four plant-based food matrices (broccoli, cashew nut, sesame, and flaxseed) to evaluate practical performance and compare against manual quantification. As shown in Figure 5, the three target lignans (sesaminol, sesamin, and sesamolin) were consistently detected across all four food matrices, and the quantitative data exhibited excellent consistency between automated detection and manual testing. This result confirmed the favorable precision of the proposed method when analyzing complex food matrices. The high-throughput automated workflow supported efficient and reliable lignan quantification in plant-based foods and thereby improved the analytical efficiency without sacrificing detection accuracy.

## 4. Conclusions

This study presents a high-throughput analytical workflow for lignan quantification in plant-derived foods, combining a Fe_3_O_4_@ZIF-67 magnetic solid-phase extraction (MSPE) with UPLC analysis. Material characterization confirmed the successful synthesis of Fe_3_O_4_ nanoparticles uniformly embedded within the ZIF-67 framework, which exhibited superparamagnetic properties, a high specific surface area, and enhanced chemical stability. Possible contributors to the observed lignan enrichment include π–π interactions, van der Waals forces, and pore-confinement effects within the porous Fe_3_O_4_@ZIF-67 framework. The material retained over 80% of its initial adsorption capacity after 60 days of storage and four consecutive reuse cycles. The results proved its outstanding reusability, structural stability, and anti-interference ability against complex food matrices, which verified its practical application potential. Method validation demonstrated excellent linearity (R^2^ ≥ 0.9999) over the concentration range of 0.5–100 μg/mL. The LOD and LOQ were in the ranges of 0.02–0.1 μg/mL and 0.09–0.25 μg/mL, respectively, and acceptable recoveries of 75.7–107.8% were obtained from spiked food samples. Comparative analysis verified that the three target lignans could be consistently identified in four food matrices (broccoli, cashew nut, sesame, and flaxseed), and the quantitative data exhibited excellent consistency between automated measurement and manual testing, which further validated the stability and dependability of the integrated detection system. Overall, this work established Fe_3_O_4_@ZIF-67 as an effective adsorbent for extracting lignans from complex food matrices and validated a high-throughput automated analytical workflow. This integrated strategy establishes an innovative technological platform for the analysis of lignans and structurally related bioactive phytochemicals in food matrices.

## Figures and Tables

**Table 1:** Linear relationship and sensitivity parameters of the proposed method.

Analytes	Linear Range (μg/mL)	R^2^	LOD (μg/mL)	LOQ (μg/mL)
Sesaminol	0.5–100	0.9999	0.10	0.25
Sesamin	0.5–100	1	0.02	0.09
Sesamolin	0.5–100	1	0.02	0.09

**Table 2:** The content, spiked recovery rate, and precision determination results of three lignan compounds in different plant-derived foods.

Sample	Analytes	Found (μg/g)	Spiked Level (μg/g)								
			3			5			7		
			Recovery (%)	Intra-Day (RSD%)	Inter-Day (RSD%)	Recovery (%)	Intra-Day (RSD%)	Inter-Day (RSD%)	Recovery (%)	Intra-Day (RSD%)	Inter-Day (RSD%)
Broccoli	Sesaminol	1.54 ± 0.32	79.9	5.1	4.9	81.2	2.8	3.3	80.5	3.1	3.6
	Sesamin	1.78 ± 0.47	85.4	6.3	5.5	86.8	1.6	1.5	85.1	6.8	7.5
	Sesamolin	1.26 ± 0.17	91.2	6.4	5.9	87.3	6.9	6.3	82.4	3.6	4.1
Cashew nut	Sesaminol	2.76 ± 0.25	78.7	8.6	8.7	82.3	2.1	1.4	84.7	2.4	0.4
	Sesamin	2.44 ± 0.37	88.4	7.3	6.8	87.2	1.8	1.2	85.4	1.4	1.3
	Sesamolin	1.95 ± 0.31	93.3	4.8	5.1	95.5	1.6	1.4	89.3	1.3	1.2
Flaxseed	Sesaminol	1.66 ± 0.24	75.7	5.9	3.2	79.3	0.8	0.9	81.3	5.3	4.1
	Sesamin	3.46 ± 0.26	83.3	13.1	13.5	85.6	1.5	0.9	91.6	3.1	7.0
	Sesamolin	3.62 ± 0.35	107.8	4.4	6.8	94.2	4.2	2.6	93.0	2.9	3.0

The results are expressed as the mean ± standard deviation (*n* = 3).

**Table 3:** Comparison of the proposed method with reported methods.

Analytes	MSPE		Bond Elut PPL		QuEChERS	
	Recovery (%)	RSD (%)	Recovery (%)	RSD (%)	Recovery (%)	RSD (%)
Sesaminol	80.65	1.32	48.87	1.50	83.62	1.65
Sesamin	83.12	2.05	39.09	1.80	89.12	1.32
Sesamolin	79.16	1.39	57.47	1.93	84.42	2.33

## Data Availability

The original contributions presented in the study are included in the article and Supplementary Materials; further inquiries can be directed to the corresponding author.

## Supplementary Materials

The following supporting information can be downloaded at: https://www.mdpi.com/article/10.3390/foods15203629/s1, Table S1. High-throughput automated extraction conditions for lignans; Table S2. Comparison of the pseudo-first-order and pseudo-second-order models for three lignans; Table S3. Parameters for the internal diffusion kinetic model of three lignans; Table S4. System adaptability verification parameters of the proposed method; Table S5. Verification parameters of the proposed method; Figure S1. Chemical structures of three lignans; Figure S2. Operation equipment diagram of the fully automatic extractor; Figure S3. The chromatogram showing the separation of 2 μg/mL lignans by a 2.7 μm C18 column. From left to right, the three chromatographic peaks are sesaminol, sesamin, and sesamolin; Figure S4. Comparison of adsorption rates of Fe_3_O_4_, Fe_3_O_4_@MOF-545, Fe_3_O_4_@MOF-545-Cys, and Fe_3_O_4_@ZIF-67 towards sesaminol, sesamin, and sesamolin; Figure S5. Adsorption kinetics of the (a) pseudo-first-order model; (b) pseudo-second-order model; (c) intra-particle diffusion for the adsorption of sesaminol, sesamin, and sesamolin on Fe_3_O_4_@ZIF-67; Figure S6. Comparison of the recovery of three lignans by automated extraction and manual operation.
