# Supplementary material for: Automated and Highly Efficient Detection of Lignans in Plant-Derived Foods Based on Magnetic Solid-Phase Extraction Combined with UPLC
Source: Foods. 2026 Oct 12;15(20):3629. doi: 10.3390/foods15203629 (PMC13703501; doi:10.3390/foods15203629)
Supplement: Supplementary file 1 [file foods-15-03629-s001.zip › foods-4582171-supplementary.pdf]

Supplementary material

Automated and Highly Efficient Detection of Lignans in Plant-Derived Foods Based on Magnetic Solid-Phase Extraction Combined with UPLC

Jiamei Zhu, Na Liang, Yang Wang and Shan Liang *

Key Laboratory of Geriatric Nutrition and Health, School of Food and Health, Beijing Technology and Business University (BTBU), Beijing 100048, China; 18238370072@163.com (J.Z.); liangna2025@163.com (N.L.); wy777800@163.com (Y.W.)

* Correspondence: liangshan@btbu.edu.cn

Contents

Table S1 .... S3

Table S2 .... S4

Table S3 .... S5

Table S4 .... S6

Table S5 .... S7

Figure S1 .... S8

Figure S2 .... S9

Figure S3 .... S10

Figure S4 .... S11

Figure S5 .... S12

Figure S6 .... S13

Table S1. High-throughput automated extraction conditions for lignans.

Step	Step name	Board position number	Mix (s)	Magnetic attraction (s)	Mixing speed	Heating operation
1	magnetic bead acquisition	1	5	60	slow	none
2	magnetic bead adsorption	2	600	60	slow	none
3	elution operation	4	360	60	slow	none
4	liquid disposal	5	0	0	slow	none

Table S2. Comparison of the pseudo-first-order and pseudo-second-order models for three lignans.

Analytes	$q_{e, exp}$ (mg/g)	Pseudo-first-order model			Pseudo-second-order model		
		k_1 (min^{-1})	$q_{e, cal}$ (mg/g)	R^2	k_2 (g/(mg·min))	$q_{e, cal}$ (mg/g)	R^2
Sesaminol	13.22	0.3121	2.01	0.9472	0.3933	13.48	0.9999
Sesamin	12.98	0.4818	4.85	0.9440	0.2268	13.33	0.9993
Sesamolin	12.81	0.4912	4.84	0.9741	0.2196	13.21	0.9994

$q_{e, exp}$ (mg/g): the equilibrium adsorption capacity predicted by this model; $q_{e, cal}$ (mg/g): experimentally determined equilibrium adsorption capacity; k_1 (min^{-1}) and k_2 (g/(mg·min)) represent the rate constants of the pseudo-first-order and pseudo-second-order kinetic models, respectively.

Table S3. Parameters for the internal diffusion kinetic model of three lignans.

**Analytes**	**k_3 (mg/(g·min$^{1/2}$))**	**I**	**R^2**
Sesaminol	0.726	11.14	0.9207
Sesamin	0.981	9.99	0.9813
Sesamolin	1.065	9.65	0.9614

k_3 (mg/(g·min$^{1/2}$)) denotes the intraparticle diffusion rate constant; I reflects the boundary layer resistance.

Table S4. System adaptability verification parameters of the proposed method.

System adaptability parameters	Analytes	Data	Judgment criteria
	Sesaminol	0.03	
Retention time (%)	Sesamin	0.02	≤ 1%
	Sesamolin	0.02	
	Sesaminol	0.21	
Peak area (%)	Sesamin	0.10	≤ 5%
	Sesamolin	0.14	
	Sesaminol	0.79	
Symmetry factor	Sesamin	0.79	≥ 0.4
	Sesamolin	0.80	

Table S5. The content, spiked recovery rate, and precision determination results of three lignan compounds in sesame.

Sample	Analytes	Found (μg/g)	Spiked level (μg/g)	Recovery (%)	Intra-day (RSD%)	Inter-day (RSD %)
Sesame	Sesaminol	12.74±1.43	20	80.3	3.6	0.3
			30	80.0	3.2	3.5
			40	80.1	6.9	4.9
	Sesamin	3675.78±25.76	3000	90.9	4.2	4.9
			4000	89.4	3.3	3.8
			5000	88.6	6.8	7.8
	Sesamolin	2043.14±17.25	1000	92.6	2.4	2.8
			2000	94.8	2.8	3.3
			3000	95.5	3.1	3.6

The results are expressed as the mean ± standard deviation (n = 3).

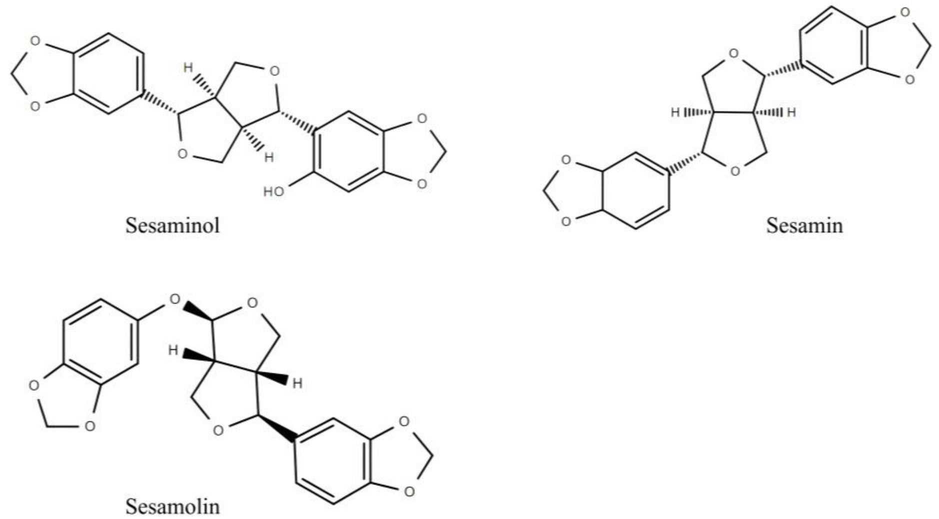

Figure S1. Chemical structures of three lignans.

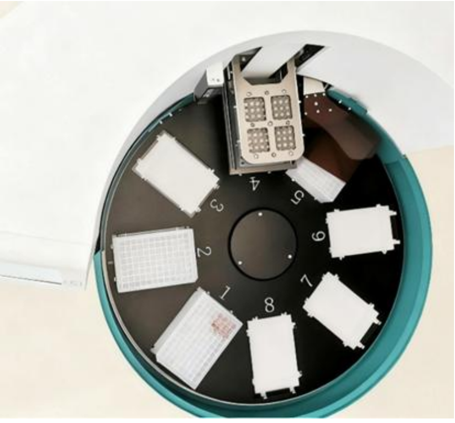

Figure S2. Operation equipment diagram of the fully automatic extractor.

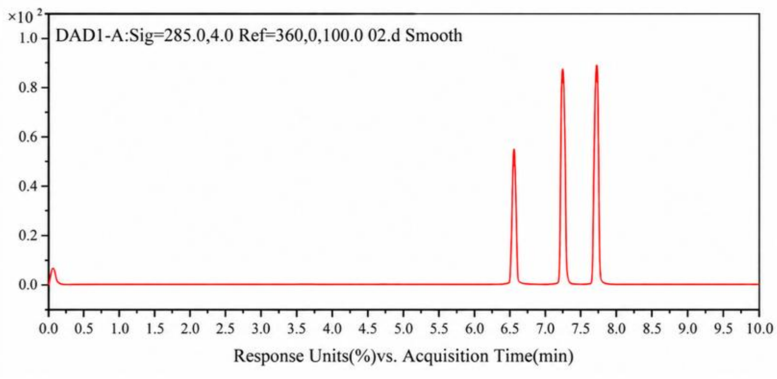

Figure S3. The chromatogram showing the separation of 2 μg/mL lignans by a 2.7 μm C18 column. From left to right, the three chromatographic peaks are sesaminol, sesamin, and sesamolin.

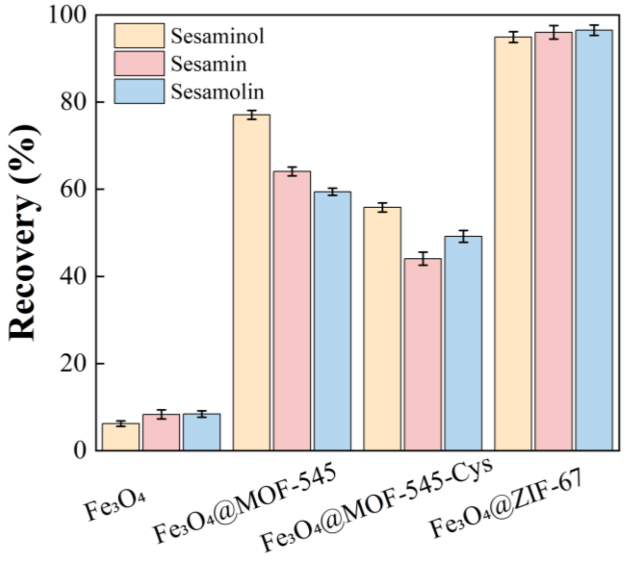

Figure S4. Comparison of adsorption rates of Fe_3O_4, Fe_3O_4@MOF-545, Fe_3O_4@MOF-545-Cys, and Fe_3O_4@ZIF-67 towards sesaminol, sesamin, and sesamolin.

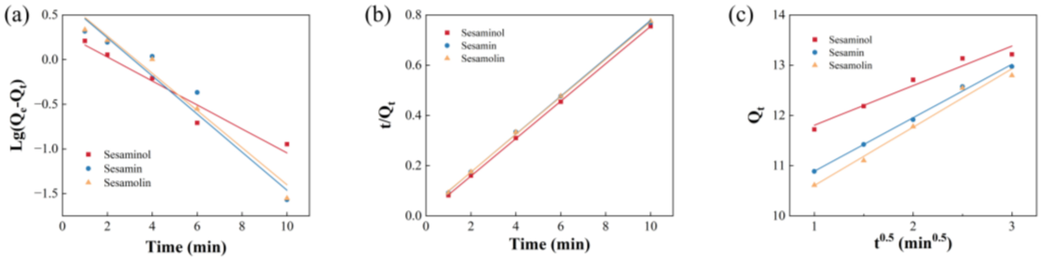

Figure S5. Adsorption kinetics of the (a) pseudo-first-order model; (b) pseudo-second-order model; (c) intra-particle diffusion for the adsorption of sesaminol, sesamin, and sesamolin on Fe_3O_4@ZIF-67.

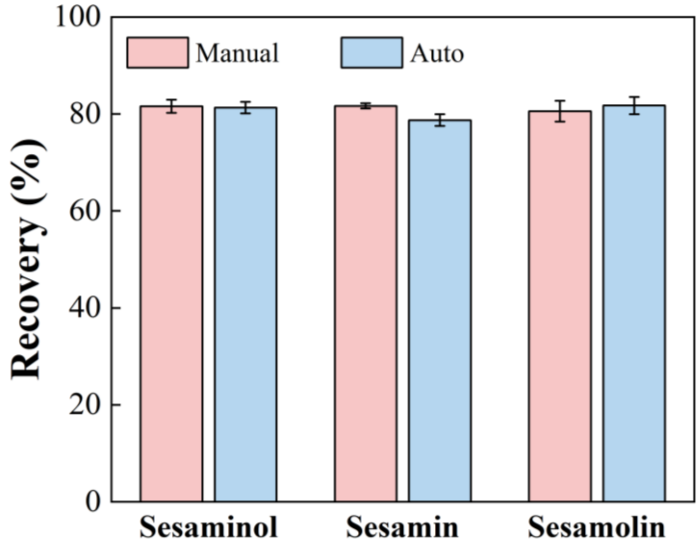

Figure S6. Comparison of the recovery of three lignans by automated extraction and manual operation.
